# Clinical outcomes of chemoradiotherapy for locally recurrent rectal cancer

**DOI:** 10.1186/1748-717X-6-51

**Published:** 2011-05-20

**Authors:** Joo Ho Lee, Dae Yong Kim, Sun Young Kim, Ji Won Park, Hyo Seong Choi, Jae Hwan Oh, Hee Jin Chang, Tae Hyun Kim, Suk Won Park

**Affiliations:** 1 Center for Colorectal Cancer, Research Institute and Hospital, National Cancer Center, Goyang, Korea; 2 Department of Radiation Oncology, Seoul National University College of Medicine, Seoul, Korea; 3 Department of Radiation Oncology, Chung-Ang University College of Medicine, Seoul, Korea

## Abstract

**Background:**

To assess the clinical outcome of chemoradiotherapy with or without surgery for locally recurrent rectal cancer (LRRC) and to find useful and significant prognostic factors for a clinical situation.

**Methods:**

Between January 2001 and February 2009, 67 LRRC patients, who entered into concurrent chemoradiotherapy with or without surgery, were reviewed retrospectively. Of the 67 patients, 45 were treated with chemoradiotherapy plus surgery, and the remaining 22 were treated with chemoradiotherapy alone. The mean radiation doses (biologically equivalent dose in 2-Gy fractions) were 54.6 Gy and 66.5 Gy for the chemoradiotherapy with and without surgery groups, respectively.

**Results:**

The median survival duration of all patients was 59 months. Five-year overall (OS), relapse-free (RFS), locoregional relapse-free (LRFS), and distant metastasis-free survival (DMFS) were 48.9%, 31.6%, 66.4%, and 40.6%, respectively. A multivariate analysis demonstrated that the presence of symptoms was an independent prognostic factor influencing OS, RFS, LRFS, and DMFS. No statistically significant difference was found in OS (p = 0.181), RFS (p = 0.113), LRFS (p = 0.379), or DMFS (p = 0.335) when comparing clinical outcomes between the chemoradiotherapy with and without surgery groups.

**Conclusions:**

Chemoradiotherapy with or without surgery could be a potential option for an LRRC cure, and the symptoms related to LRRC were a significant prognostic factor predicting poor clinical outcome. The chemoradiotherapy scheme for LRRC patients should be adjusted to the possibility of resectability and risk of local failure to focus on local control.

## Background

Recent advances in preoperative evaluation, treatment strategies and rectal cancer modalities have lead to better survival outcomes for patients with rectal cancer and a lower incidence of local recurrence [1,2]. Despite such improvements, 6-10% of patients with primary rectal cancer still experience intrapelvic local recurrence with or without distant metastasis [3-5]. These patients show a poor survival outcome with a nearly zero 5-year survival and 3-12 months of median survival when treated by only supportive care or palliative treatment [4]. Moreover, troublesome symptoms related to local recurrence reduce the quality of life during surviving periods. Recent studies have reported that radical surgery with microscopic curative resection presents a 48-60% long-term survival rate in patients surviving at 5 years [3,4,6-9]. These observations suggest that local control of LRRC is significantly associated with long-term survival and that the first goal of LRRC treatment should be local tumor control [5].

However, an aggressive approach with surgery alone also has severe weaknesses in that curative surgery is possible for only 20-30% of patients with locally recurrent rectal cancer (LRRC), because the intrapelvic space is too narrow to perform an R0 resection, and previous treatments, including surgery and radiotherapy, induce extensive fibrosis [3,4]. Moreover, high post-operative morbidities, of 30-60% [6-8], and the non-operable state of some patients should also be considered in the clinical situation. To compensate for the shortage of radical surgery, chemoradiotherapy (CRT) with adjuvant or curative intent has a definitive role in improving the clinical outcome of patients with LRRC. Some studies have demonstrated that multimodal treatment including CRT results in better clinical outcomes, but the role and strategies for CRT have not yet been established. Thus, the purpose of the present study was to assess the clinical outcomes of CRT with or without surgery for patients with LRRC and to find useful and significant prognostic factors for the clinical situation.

## Methods

### Patients

This study was performed in accordance with the guidelines of our institutional review board. All patients provided written informed consent before salvage treatment.

Between January 2001 and February 2009, 67 patients with LRRC underwent CRT with or without surgery as a salvage treatment at the National Cancer Center (Goyang, Korea). Inclusion criteria were: *(1) *histologically confirmed primary rectal adenocarcinoma, *(2) *recurrent sites confined to the pelvic cavity, *(3) *no evidence of distant metastasis, and *(4) *salvage treatment with a curative aim.

Patient characteristics are shown in Table 1. The recurrence-free interval from the initial treatment of the primary tumor to locoregional recurrence ranged from 3 to 206 months (median, 22 months). Of 67 patients, 45 (67.2%) presented with local recurrence after a sphincter-saving radical surgery to remove a primary tumor, 17 patients (25.4%) developed recurrence following an abdominoperineal resection, and five patients (7.5%) experienced recurrence following local excision. Fifty-five patients (82.1%) had a history of adjuvant chemotherapy for a primary tumor, and 23 (34.3%) received adjuvant radiotherapy for a primary tumor. Symptoms related to local recurrence were sciatic pain in 17 patients, bowel habit changes in two patients, and a ureteral obstruction in one patient.

**Table 1:** Patient and treatment characteristics

Characteristics	Value (%)
Median age, years (range)	57 (30-84)
Gender	
Male	40 (59.7)
Female	27 (40.3)
Stage at initial diagnosis	
ypStage 0	3 (4.2)
pStage I/ypStage I	5 (7.0)/1 (1.4)
pStage II/ypStage II	14 (19.7)/3 (4.2)
pStage III/ypStage III	21 (29.6)/8 (11.3)
pStage IV/ypStage IV	6 (8.5)/1 (1.4)
pT1-2Nx	5 (7.0)
Recurrence history	
0	51 (76.1)
1	16 (23.9)
Symptoms at recurrence	
Yes	20 (29.9)
No	47 (70.1)
Recurrent site	
Central	21 (31.3)
Lateral	30 (44.8)
Posterior	16 (23.9)
Pretreatment CEA	
Normal (≤ 5 ng/mL)	37 (55.2)
High (> 5 ng/mL)	30 (44.8)
Salvage treatment	
Surgery + CRT	45 (67.2)
CRT alone	22 (32.8)
Chemotherapy regimen	
Fluoropyrimidine-alone	35 (52.2)
Irinotecan or Oxaliplatin-based	31 (46.3)
No	1 (1.5)
Radicality of resection	
R0	19 (28.4)
R1	24 (35.8)
R2	2 (4.0)
No surgery	22 (32.8)
Median radiation dose, Gy (range)	57.2 (44.3-74.4)

Values in parentheses are percentages unless indicated otherwise. CRT, chemoradiotherapy; R0, microscopically radical; R1, microscopically irradical; R2, macroscopically irradical; Gy, Gray; CEA, carcinoembryonic antigen.

Through biopsy or surgical resection, 45 patients were confirmed histologically to have developed a local recurrence. In 22 patients, radiological evidence, including a positive positron-emission tomography (PET) scan or serial radiological examinations that showed progressive growth of the mass, were considered sufficient evidence to diagnose a local recurrence [10,11]. All patients were evaluated by digital rectal examination, a complete blood count, a liver function test, carcinoembryonic antigen (CEA) level, computed tomography (CT) of the chest and abdomino-pelvis, whole body PET, and magnetic resonance imaging (MRI) of the pelvis.

### Treatment

Following the diagnosis of a locoregional recurrence, a surgeon, a medical oncologist, and a radiation oncologist reviewed the results of the diagnostic work-up to determine which treatment modality would be best suited for each patient. Considered unsuitable for curative surgery, 22 patients among 67 patients received definitive CRT without surgery. The other 45 underwent resection of a locally recurrent lesion with curative intent and preoperative (n = 3) or postoperative CRT (n = 42). Most adjuvant RT approaches consisted of post-operative, rather than pre-operative, as following reasons. *(1) *If a diagnosis is uncertain, histological confirm was possible through surgery. *(2) *If a patient has limitations for RT, such as previous RT history or small bowel adhesion at recurrence site, we performed omental flap transposition [12]. It functioned as spacer to increase a distance between small bowel and RT target area. *(3) *If RT target area and rectum are too close, we could perform protective colostomy for the prevention of RT-induced proctitis. *(4) *In some cases, preferences of doctor and patient were cause of such practice.

Radiotherapy was administered using three-dimensional conformal radiation (*n *= 60), proton beam therapy (*n *= 4), or helical tomotherapy (*n *= 3). All patients underwent a CT simulation in the treatment position, which was generally prone. The gross tumor volume, consisting of all detectable tumors, was determined from the CT, PET, and MRI data. The clinical target volume covered the gross tumor volume, tumor bed, and other suspicious microscopic lesions. The initial planning target volume included the clinical target volume plus a 10-20 mm margin. Organs at risk were also delineated, including the spinal cord, bladder, both kidneys, and the small bowel.

The radiation dose was 45-72 Gy, with fraction sizes of 1.8-3.0 Gy (biologically equivalent dose in 2-Gy fractions [BED_2Gy_] using a linear quadratic model, and the α/β; ratio was 10 for acute effects on normal tissues and tumors: 44.3-74.4 BED_2Gy_), and the median dose was 57.2 BED_2Gy_. The dose-fractionation schedules were as follows: 1.8 Gy/fraction in 60 patients, 2.4 Gy/fraction in four patients, 2.7 Gy/fraction in one patient, 2.8 Gy/fraction in one patient, and a 3 Gy/fraction in one patient. The radiation dose was adjusted according to the status of the residual tumor, radiation history, and proximity to the small bowel.

Most patients underwent concurrent chemotherapy with radiation, consisting of a fluoropyrimidine (*n *= 35), irinotecan, or oxaliplatin-based regimens (*n *= 31). Only one patient could not receive chemotherapy, because of hepatitis. Maintenance chemotherapy after concurrent CRT was applied to 88.1% of patients (*n *= 59), which consisted of a fluoropyrimidine regimen (*n *= 23) and an irinotecan or oxaliplatin-based regimen (*n *= 36). The remaining eight patients did not undergo maintenance chemotherapy because of patient refusal (*n *= 6) or poor performance status (*n *= 2).

### Evaluation

After salvage treatment, follow-up was performed every 3 months for the first 2 post-treatment years and every 6 months thereafter. Follow-up evaluations included a physical examination, digital rectal examination, complete blood count, liver function test, and serum CEA level at each visit. Chest radiography and CT scanning of the abdomen and pelvis were performed every 6 months after salvage treatment. Relapse after salvage treatment was confirmed pathologically by direct biopsy or cytology, and/or radiographical evidence. Locoregional failure was defined as a new lesion or disease progression within the pelvic cavity, and distant failure as any recurrence outside the pelvic cavity.

### Statistical Analyses

Overall survival (OS), relapse-free survival (RFS), locoregional relapse-free survival (LRFS), and distant metastasis-free survival (DMFS) were calculated as the interval from the first date of salvage treatment to the date of death, any relapse detection, locoregional relapse detection, or distant metastasis detection, respectively.

Survival curves were generated by the Kaplan-Meier method, and a univariate survival comparison was performed using the log-rank test. Multivariate analyses were conducted with the Cox proportional hazards model and the backward stepwise selection procedure. The chi-squared, Fisher's exact, and *t*-tests were performed to compare various parameters between different treatment groups. A p-value of <  0.05 was considered to indicate statistical significance.

## Results

### Survival and pattern of failure

The median follow-up time for living patients was 41 months (range, 16-108). The median OS of all patients was 59 months. Median RFS, LRFS, and DMFS were 18, not reached, and 23 months, respectively. Five-year OS, RFS, LRFS, and DMFS were 48.9%, 31.6%, 66.4%, and 40.6%, respectively. A relapse after salvage treatment occurred in 41 (61.2%) patients during the follow-up period, and locoregional failure was detected in six patients (9.0%), distant metastasis in 30 patients (44.8%), and both failures in five patients (7.5%). During follow-up period, severe G-I complication over Grade III, associated with CRT, did not occur.

### Analysis of prognostic factors

The univariate analysis of the effect of prognostic factors on clinical outcome is shown in Table 2. The presence of symptoms was a significant prognostic factor correlated with poor OS (p = 0.025), RFS (p = 0.007), LRFS (p = 0.003), and DMFS (p = 0.047). In contrast, age, gender, type of primary surgery, recurrence-free interval, recurrence history, recurrence site, pre-treatment CEA serum level, salvage treatment, chemotherapy regimen, resection margin, and radiation dose had no statistically significant effect on OS, RFS, LFS, or DMFS. In the multivariate analysis, the presence of symptoms was an independent prognostic factor predicting poor OS (p = 0.025; hazard ratio [HR], 3.46; 95% confidence interval [CI], 1.17-10.22), RFS (p = 0.017; HR, 3.04; 95% CI, 1.22-7.59), LRFS (p = 0.005; HR, 3.60; 95% CI, 1.48-8.80), and DMFS (p = 0.032; HR, 2.93; 95% CI, 1.10-7.89).

**Table 2:** Univariate analysis of factors affecting clinical outcome

	5y OS	p†	5y RFS	p†	5y LRFS	p†	5y DMFS	p†
Age (years)								
< 60	50.5	.653	27.6	.547	57.6	.084		
≥60	47.9		36.2		75.9			
Gender								
Male	55.6	.381	34.8	.340	73.7	.141	42.3	.811
Female	34.9		27.7		55.7		38.1	
Recurrence-free interval (months)								
<24	46.4	.675	33.2	.473	59.8	.248	40.6	.663
≥24	50.7		26.9		72.9		38.5	
Previous recurrence history								
0	51.2	.417	36.4	.061	66.9	.834	43.0	.420
1	38.8		16.7		65.2		33.0	
Symptoms at recurrence								
Yes	26.3	.025	20.0	.007	40.0	.003	25.8	.047
No	58.4		38.3		76.2		47.2	
Recurrence site								
Central	56.0	.494	43.5	.429	67.7	.918	54.2	.305
Lateral	44.8		26.0		69.1		31.5	
posterior	36.4		35.3		63.5		36.2	
Pretreatment CEA (ng/mL)								
≤5	45.7	.882	41.8	.071	72.1	.154	49.2	.458
>5	52.8		21.8		59.1		33.8	
Salvage Treatment								
Surgery + CRT	52.8	.181	35.2	.113	71.0	.379	43.6	.335
CRT alone	40.6		24.5		55.9		34.6	
Chemotherapy regimen								
Fluoropyrimidines-alone	47.3	.910	36.7	.572	67.2	.720	42.9	.562
Irinotecan or Oxaliplatin -based	41.0		22.0		64.7		33.8	
Resection§								
R0	60.4	.994	35.1	.956	77.7	.529	37.6	.919
R1 or R2	42.9		34.3		65.6		46.8	
Radiation dose (BED_2Gy_)								
<60	46.9	.607	32.3	.281	78.2	.065	41.0	.694
≥60	48.3		29.2		52.9		39.2	

*values are percentages of patients; †log rank test. OS, overall survival; **§ **Among 45 patients undergoing surgical resection; RFS, recurrence-free survival; LRFS, locoregional relapse free survival; DMFS, distant metastasis-free survival; CRT, chemoradiotherapy; R0, microscopically radical; R1, microscopically irradical; R2, macroscopically irradical; BED_2Gy_, biologically equivalent dose in 2-Gy fractions using a linear quadratic model, and the α/β ratio was 10 for acute effects on normal tissues and tumors. CEA, carcinoembryonic antigen.

### Comparison between CRT with and without surgery

**Figure 1:**
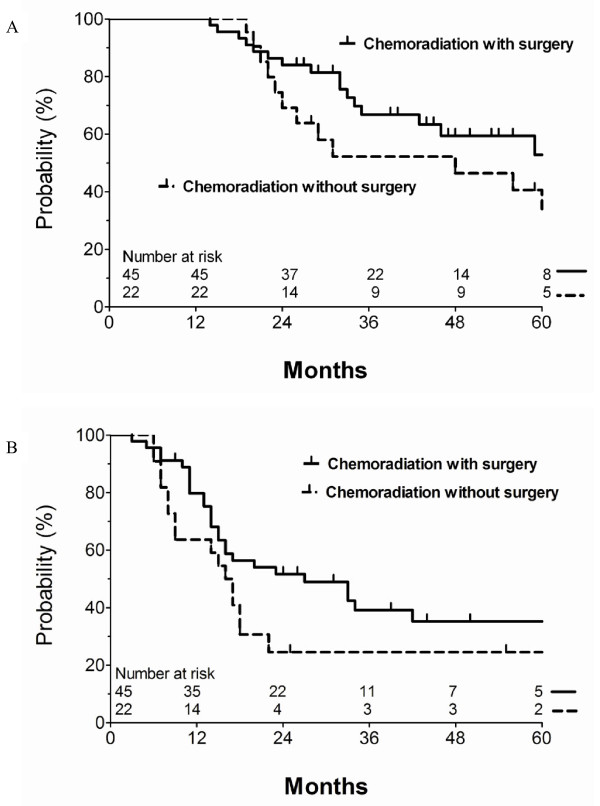
**Overall survival (a) and relapse-free survival (b) between the chemoradiotherapy with surgery and without surgery groups**.

No statistically significant difference was found in OS (p = 0.181), RFS (p = 0.113), LRFS (p = 0.379), or DMFS (p = 0.458) when clinical outcomes were compared between the CRT with surgery and definitive CRT without surgery groups. Figure 1 shows the OS and RFS curves for each group. The prognostic factors, as described above, were stratified by the two groups and are shown in Table 3. Significantly more patients with symptoms and an abnormal CEA level (> 5 ng/mL) received definitive CRT without surgery (p = 0.014, 0.009, respectively). The mean radiation dose was 54.6 BED_2Gy _in the CRT with surgery group, and 66.5 BED_2Gy _in the definitive CRT without surgery group (p < 0.001). In addition, post-operative RT dose was also different according to margins status. Patients with a positive resection margin received the higher radiation dose (mean dose, 57.5 BED2Gy) than patients with a negative resection margin (mean dose, 50.6 BED2Gy).

**Table 3:** Patient characteristics between the surgery plus chemoradiation and chemoradiation alone groups

Characteristic	Surgery + chemoradiation (*n *= 45)	chemoradiation (*n *= 22)	P
Mean age, years	56.7 ± 11.5	60.0 ± 13.4	0.377§
Gender			
Male	25	15	0.322†
Female	20	7	
Recurrence-free interval, months	35.3	30.0	0.521§
Chemotherapy history			
Yes	35	20	0.310‡
No	10	2	
Radiation history			
Yes	14	6	0.747†
No	31	16	
Recurrence history			
0	35	16	0.649†
1	10	6	
Symptoms at recurrence			
Yes	9	11	0.014†
no	36	11	
Recurrence site			
Central	16	5	0.440†
Lateral	20	10	
posterior	9	7	
Pretreatment CEA (ng/mL)			
≤5	30	7	0.009†
>5	15	15	
Chemotherapy regimen			
Fluoropyrimidines alone	21	13	0.249†
Irinotecan or oxaliplatin-based	24	8	
Radiation dose (BED_2Gy_)			
<60	34	1	<0.001†
≥60	11	21	
Mean radiation dose, BED_2Gy_	54.6 ± 5.5	66.5 ± 6.2	<0.001§

†chi-squared test; ‡ Fisher exact test; **§ **t-test; BED_2Gy_, biologically equivalent dose in 2-Gy fractions using a linear quadratic model, CEA, carcinoembryonic antigen; and the α/β; ratio was 10 for acute effects on normal tissues and tumors.

## Discussion

This study assessed whether CRT with or without surgery was effective in patients with LRRC and identified useful prognostic factors for the clinical setting. A 5-year OS of 48.9% and a LRFS of 66.4% was achieved; this outcome was better than previous multimodal treatment reports (5-yr OS of 25-36%, LRFS of 40-50%). However, DMFS was similar to the results of previous studies and was approximately 40-50% [6-8,13-15]. When evaluating prognostic factors, symptoms related to LRRC have a significant effect on OS, RFS, LRFS, and DMFS. Pretreatment quality of life could be related to the clinical outcomes for many kinds of cancer and could be considered a potential prognostic factor. In other studies, symptoms related to LRRC have been reported as significant prognostic factors for a poor outcome [6,7,13] and such patients are considered a low possibility for radical resection [13]. Hydronephrosis presenting in two patients indicated a lower chance for obtaining a negative resection margin [16]. LRRC symptoms are a useful and readily assessable prognostic factor in the clinical setting.

Another strength of the present study was that CRT was tailored to the individual risk of a residual tumor and the potential risk of a complication after an attempt at curative resection. The patients in the LRRC group were actually heterogeneous when considering resectability, the strongest factor affecting clinical outcome. Tumor location and the degree of local invasion affect resectability, and the posterior and lateral location, particularly including a sacral, ureteral, or iliac vessel invasion, are almost unresectable and cause marked postoperative disability [10,17]. Many studies on multimodal LRRC treatment have attempted preoperative CRT to solve the problem of low resectability [6-9,13,18-20], and one of those studies demonstrated significantly increased resectability [6]. However, resectability improved by preoperative CRT was still insufficient, at 30-60% [6-8,13,15,18,21]. The remaining 40-70% of patients with incompletely resected LRRC showed disappointing local control (30% 3-year LRFS), and this insufficient local control lead to a poor survival outcome of 10-16% for the 5-year OS [6,8]. Moreover, the pre-operative CRT radiation dose was a uniform low dose of 30-50 Gy, but did not consider the risk of an unresectable or residual tumor. When local control is the prime goal of LRRC treatment, the radiation dose or CRT plan should be determined based on such risks for local failure and complication.

All patients, except three who underwent preoperative CRT followed by radical resection, received CRT with an adjusted postoperative or definitive radiation dose, based on the risk for local failure and complication. In the preoperative evaluation, poor surgical candidates who were definitively unresectable or medically inoperable underwent definitive CRT with a high radiation dose (mean dose, 66.5 BED_2Gy_). In patients with a positive resection margin, the post-operative radiation dose (mean dose, 57.5 BED_2Gy_) was also higher than in patients with a negative resection margin (mean dose, 50.6 BED_2Gy_). Some studies have demonstrated that a higher radiation dose for patients with LRRC is correlated with better clinical outcome [6,20]. Fifteen patients underwent omental flap transposition as a spacer, as proposed by Kim et al. [12] and seven patients received proton beam or helical tomotherapy to safely deliver a high dose of radiation to recurrent sites in patients who had previously undergone radiation and whose small bowel is very close to the target area. The radiation plan also focused on risky areas for local failure, referring to operative findings and pathological reports. As a result, this study showed improved local control, leading to improved OS. Moreover, patients with a positive resection margin demonstrated notably better outcomes (5-year OS, 42.9%) than other studies [6,7,13]. This study showed that the purpose of CRT should not be just adjuvant, aimed at increasing resectability, but an aggressive curative local control, similar to surgery. Such a treatment plan could result in an increased cure rate with long-term survival.

The present study also showed that definitive CRT with a high radiation dose (mean dose, 66.5 BED_2Gy_) may be a potentially curative option for long-term survival (5-year OS, 48.9%). The actuarial 5-year OS, RFS, LRFS, and DMFS for definitive CRT was not significantly different than CRT with surgery. However, median OS, RFS, LRFS, and DMFS for definitive CRT tended to be slightly inferior to the surgery group, but this difference was not statistically significant. Patients with an abnormal CEA level or the presence of symptoms occurred more in the definitive CRT group, and this may have affected the outcome of the definitive CRT group. Symptoms were a significant prognostic factor in the present study and CEA level has been reported as a significant prognostic factor in some previous studies [22,23]. Although definitive CRT cannot substitute for radical surgery, it can be an option aimed at a cure with long-term survival for a fair number of patients with an inoperable medical condition or an unresectable lesion.

The present study has some limitations. First, in contrast to other studies, the radicality of resection was not a significant prognostic factor predicting survival outcome or tumor control. It might be related with low statistical power due to small sample size (*n *= 67). In addition, the reason could be also that radiation dose was increased according to residual tumor status. Such a difference in the radiation dose appeared to dilute the effect of surgical radicality. Another reason could be that a relatively small proportion of R2 resections (4%) of the CRT with surgery might induce improvement in the group with positive resection margin. Patients with expected unresectability from the radiological evaluation were recommended for definitive CRT without surgery, so a R2 resection might have been rarer than in other studies. In that R2 resection have more effect on an unfavorable clinical outcome than R1 resection [24], the effect of radicality might fail to get the statistical significance. Second, we could observe tendency in the survival curves that the CRT with surgery got the slightly more favorable outcome than the definitive CRT group, but it failed to get a statistical significance. This could be resulted from the effects of a small sample size, surgical morbidities, and the differences of radiation dose. This study showed the possibility of a definitive CRT for cure, but further study with a larger sample size is needed for a definitive conclusion about the comparison between the two groups. Third, we had a heterogeneous population undergoing different CRT approaches and chemotherapy regimens. Accordingly, further larger scale and prospective studies with additional long-term follow-up are needed to compare different CRT approaches definitively.

## Conclusions

Our study demonstrated that LRRC has the potential to be cured with CRT with or without surgery, and the symptoms related to LRRC are a significant prognostic factor predicting poor clinical outcome. The CRT approach should focus on local control; thus, individualized CRT strategies are recommended, based on the possibility of resectability and risk of local failure. Thus, CRT with an adjusted radiation dose is a potential curative option for LRRC, including definitive CRT without surgery.

## Competing interests

The authors declare that they have no competing interests.

## Authors' contributions

DYK contributed to conception and design of the study, and revised the manuscript. JHL, SYK, JWP, and THK contributed to analysis and interpretation of data, and drafted the manuscript. HJC, HSC participated in revising the manuscript. JHO participated in data acquisition and literature research. SWP contributed to conception of the study. All authors read and approved the final manuscript.

## References

[B1] Colorectal Cancer Collaborative Group Adjuvant radiotherapy for rectal cancer: a systematic overview of 8,507 patients from 22 randomised trials Lancet 2001 358 1291 1304 11684209 10.1016/S0140-6736(01)06409-1

[B2] Heald RJ Moran BJ Ryall RD Sexton R MacFarlane JK Rectal cancer: the Basingstoke experience of total mesorectal excision, 1978-1997 Arch Surg 1998 133 894 899 10.1001/archsurg.133.8.894 9711965

[B3] Bakx R Visser O Josso J Meijer S Slors JF van Lanschot JJ Management of recurrent rectal cancer: a population based study in greater Amsterdam World J Gastroenterol 2008 14 6018 6023 10.3748/wjg.14.6018 18932280 PMC2760194

[B4] Palmer G Martling A Cedermark B Holm T A population-based study on the management and outcome in patients with locally recurrent rectal cancer Ann Surg Oncol 2007 14 447 454 10.1245/s10434-006-9256-9 17139457

[B5] Kim TH Chang HJ Kim DY Jung KH Hong YS Kim SY Park JW Oh JH Lim SB Choi HS Jeong SY Pathologic nodal classification is the most discriminating prognostic factor for disease-free survival in rectal cancer patients treated with preoperative chemoradiotherapy and curative resection Int J Radiat Oncol Biol Phys 2010 77 1158 1165 10.1016/j.ijrobp.2009.06.019 19800178

[B6] Dresen RC Gosens MJ Martijn H Nieuwenhuijzen GA Creemers GJ Daniels-Gooszen AW van den Brule AJ van den Berg HA Rutten HJ Radical resection after IORT-containing multimodality treatment is the most important determinant for outcome in patients treated for locally recurrent rectal cancer Ann Surg Oncol 2008 15 1937 1947 10.1245/s10434-008-9896-z 18389321 PMC2467498

[B7] Hahnloser D Nelson H Gunderson LL Hassan I Haddock MG O'Connell MJ Cha S Sargent DJ Horgan A Curative potential of multimodality therapy for locally recurrent rectal cancer Ann Surg 2003 237 502 508 12677146 10.1097/01.SLA.0000059972.90598.5F PMC1514480

[B8] Heriot AG Byrne CM Lee P Dobbs B Tilney H Solomon MJ Mackay J Frizelle F Extended radical resection: the choice for locally recurrent rectal cancer Dis Colon Rectum 2008 51 284 291 10.1007/s10350-007-9152-9 18204879

[B9] Kusters M Dresen RC Martijn H Nieuwenhuijzen GA van de Velde CJ van den Berg HA Beets-Tan RG Rutten HJ Radicality of resection and survival after multimodality treatment is influenced by subsite of locally recurrent rectal cancer Int J Radiat Oncol Biol Phys 2009 75 1444 1449 10.1016/j.ijrobp.2009.01.015 19395199

[B10] Bouchard P Efron J Management of recurrent rectal cancer Ann Surg Oncol 2010 17 1343 1356 10.1245/s10434-009-0861-2 20041351

[B11] Watson AJ Lolohea S Robertson GM Frizelle FA The role of positron emission tomography in the management of recurrent colorectal cancer: a review Dis Colon Rectum 2007 50 102 114 10.1007/s10350-006-0735-7 17115340

[B12] Kim TH Kim DY Jung KH Hong YS Kim SY Park JW Lim SB Choi HS Jeong SY Oh JH The role of omental flap transposition in patients with locoregional recurrent rectal cancer treated with reirradiation J Surg Oncol 2010 102 789 95 10.1002/jso.21737 20886555

[B13] Pacelli F Tortorelli AP Rosa F Bossola M Sanchez AM Papa V Valentini V Doglietto GB Locally recurrent rectal cancer: prognostic factors and long-term outcomes of multimodal therapy Ann Surg Oncol 2010 17 152 162 10.1245/s10434-009-0737-5 19834766

[B14] Wiig JN Larsen SG Giercksky KE Operative treatment of locally recurrent rectal cancer Recent Results Cancer Res 2005 165 136 147 10.1007/3-540-27449-9_15 15865028

[B15] Valentini V Morganti AG Gambacorta MA Mohiuddin M Doglietto GB Coco C De Paoli A Rossi C Di Russo A Valvo F Bolzicco G Dalla Palma M Study Group for Therapies of Rectal Malignancies (STORM) Preoperative hyperfractionated chemoradiation for locally recurrent rectal cancer in patients previously irradiated to the pelvis: A multicentric phase II study Int J Radiat Oncol Biol Phys 2006 64 1129 1139 10.1016/j.ijrobp.2005.09.017 16414206

[B16] Larsen SG Wiig JN Giercksky KE Hydronephrosis as a prognostic factor in pelvic recurrence from rectal and colon carcinomas Am J Surg 2005 190 55 60 10.1016/j.amjsurg.2004.07.043 15972173

[B17] Park JK Kim YW Hur H Kim NK Min BS Sohn SK Choi YD Kim YT Ahn JB Roh JK Keum KC Seong JS Prognostic factors affecting oncologic outcomes in patients with locally recurrent rectal cancer: impact of patterns of pelvic recurrence on curative resection Langenbecks Arch Surg 2009 394 71 77 10.1007/s00423-008-0391-6 18663464

[B18] Wiig JN Tveit KM Poulsen JP Olsen DR Giercksky KE Preoperative irradiation and surgery for recurrent rectal cancer. Will intraoperative radiotherapy (IORT) be of additional benefit? A prospective study Radiother Oncol 2002 62 207 213 10.1016/S0167-8140(01)00486-8 11937248

[B19] Saito N Koda K Takiguchi N Oda K Ono M Sugito M Kawashima K Ito M Curative surgery for local pelvic recurrence of rectal cancer Dig Surg 2003 20 192 199 10.1159/000070385 12759498

[B20] Mohiuddin M Marks G Marks J Long-term results of reirradiation for patients with recurrent rectal carcinoma Cancer 2002 95 1144 1150 10.1002/cncr.10799 12209702

[B21] Schurr P Lentz E Block S Kaifi J Kleinhans H Cataldegirmen G Kutup A Schneider C Strate T Yekebas E Izbicki J Radical redo surgery for local rectal cancer recurrence improves overall survival: a single center experience J Gastrointest Surg 2008 12 1232 1238 10.1007/s11605-008-0517-8 18446418

[B22] Asoglu O Karanlik H Muslumanoglu M Igci A Emek E Ozmen V Kecer M Parlak M Kapran Y Prognostic and predictive factors after surgical treatment for locally recurrent rectal cancer: a single institute experience Eur J Surg Oncol 2007 33 1199 1206 17400423 10.1016/j.ejso.2007.02.026

[B23] Bedrosian I Giacco G Pederson L Rodriguez-Bigas MA Feig B Hunt KK Ellis L Curley SA Vauthey JN Delclos M Crane CH Janjan N Skibber JM Outcome after curative resection for locally recurrent rectal cancer Dis Colon Rectum 2006 49 175 182 10.1007/s10350-005-0276-5 16392024

[B24] Suzuki K Gunderson LL Devine RM Weaver AL Dozois RR Ilstrup DM Martenson JA O'Connell MJ Intraoperative irradiation after palliative surgery for locally recurrent rectal cancer Cancer 1995 75 939 952 10.1002/1097-0142(19950215)75:4<939::AID-CNCR2820750408>3.0.CO;2-E 7531113

